# Targeted Antitumor Mechanism of C-PC/CMC-CD55sp Nanospheres in HeLa Cervical Cancer Cells

**DOI:** 10.3389/fphar.2020.00906

**Published:** 2020-06-18

**Authors:** Guoxiang Liu, Xiaohui Xu, Liangqian Jiang, Huanhuan Ji, Feng Zhu, Bingnan Jin, Jingjing Han, Xiaolei Dong, Fanghao Yang, Bing Li

**Affiliations:** ^1^Department of Genetics and Cell Biology, Basic Medical College, Qingdao University, Qingdao, China; ^2^Department of Medical Genetics, Linyi People's Hospital, Linyi, China; ^3^Department of Hematology, The Affiliated Hospital of Qingdao University, Qingdao, China

**Keywords:** C-phycocyanin, carboxymethyl chitosan, CD55 ligand peptide, targeting nanospheres, HeLa cells

## Abstract

*In vitro* studies had shown that C-Phycocyanin (C-PC) inhibited cervical cancer HeLa cells growth. We constructed C-PC/CMC-CD55sp nanospheres using C-PC, Carboxymethyl Chitosan (CMC), and CD55 ligand peptide (CD55sp) to allow for targeted antitumor effects against HeLa cells *in vitro* and *in vivo*. The characteristics of the nanospheres were determined using FTIR, electron microscopy, and laser particle size analysis. Flow cytometry, laser confocal microscopy and small animal imaging system showed the targeting of C-PC/CMC-CD55sp nanospheres on HeLa cells. Subsequently, the proliferation and apoptosis were analyzed by Cell Counting Kit-8 (CCK-8), flow cytometry, TUNEL assay and electron microscopy. The expression of the apoptosis-related protein was determined using western blot. The stainings of Hematoxylin and Eosin (HE) were employed to evaluate the cell condition of tumor tissue sections. The cytokines in the blood in tumor-bearing nude mice was determined using ELISA. These results showed that C-PC/CMC-CD55sp nanospheres were successfully constructed and targeted HeLa cells. The constructed nanospheres were more effective than C-PC alone in inhibiting the proliferation and inducing apoptosis in HeLa cells. We also found that C-PC/CMC-CD55sp nanospheres had a significant inhibitory effect on the expression of antiapoptotic protein Bcl-2 and a promotion on the transformation of caspase 3 to cleaved caspase 3. C-PC/CMC-CD55sp nanospheres played an important role in tumor suppression, reduced the expression TGF-β, and increased IL-6 and TNF-α. This study demonstrates that the constructed new C-PC/CMC-CD55sp nanospheres exerted targeted antitumor effects *in vivo* and *in vitro* which provided a novel idea for application of C-PC, and provided experimental basis for comprehensive targeted treatment of tumors.

## Introduction

Early screening and vaccination are the most common methods to prevent cervical cancer (Torre et al., 2015), and chemotherapy is a first-line treatment option for patients with cervical cancer. Cetuximab combined with carboplatin and paclitaxel can effectively treat advanced/recurrent cervical cancer (Pignata et al., 2019). Pembrolizumab monotherapy has also been shown to exhibit lasting antitumor activity with adequate safety in patients with advanced cervical cancer (Chung et al., 2019). However, due to lack of targeting, chemotherapy drugs also affect normal cells (Cao et al., 2018). Tumor-targeted drug delivery systems represent an important advancement in cancer therapy. These formulations can deliver effective antitumor drugs specifically or selectively to tumor tissues, which allows for control of drug dosing at specific physiological sites. Specific targeting results in reduced side effects and toxicity at nontargeted sites (Schellmann et al., 2010; Chow et al., 2011; Liu F. et al., 2014; Costa Lima et al., 2017). Nanospheres have been conjugated to a monoclonal antibody against Trophoblast cell surface antigen 2 (TROP2), which is a protein abundant on the surface of HeLa cells. This formulation selectively killed cervical cancer cells through induction of apoptosis and DNA damage (Liu T. et al., 2014). Magnetically responsive bacterial polyester-based nanospheres that encapsulating etoposide and modified with concanavalin-A have been used to target cervical cancer (HeLa) cells (Erdal et al., 2012). Folic acid–conjugated albumin nanospheres have been developed to target drugs to cervical cancer cells, and to reduce side effects (Shen et al., 2011).

Rapid development of nanotechnology has allowed for construction of drug-chitosan nanospheres (Azab et al., 2007; Zhang et al., 2008). C-PC (Zheng et al., 2013), a natural photosynthetic pigment, has antiaging and antioxidative effects, and is nontoxic, safe, and water-soluble. C-PC fluoresces red, and can be used as a fluorescent marker. Li et al. (Li et al., 2010; Li et al., 2016) find that C-PC inhibits tumor growth. However, C-PC is rapidly degraded by proteases *in vivo*, which has limited its use in the pharmaceutical industry. Chitosan (Xu and Du, 2003; Narayanan et al., 2014; Snima et al., 2014; Ravindranathan et al., 2016; Qi et al., 2018) is a natural polymer material that is nontoxic, biocompatible, and biodegradable, and exerts antibacterial, antiinflammatory, wound healing, and antitumor effects. Use of chitosan has been limited by poor water solubility. CMC (Snima et al., 2012; Farag and Mohamed, 2012; Kumar Singh Yadav and Shivakumar, 2012; Maya et al., 2013; Sharif et al., 2017), formed by carboxylation of chitosan, exhibits good water solubility and biocompatibility, is nontoxic, and promotes bacteriostasis (Jiang et al., 2004). CMC has been used as a drug carrier to improve drug efficacy, reduce side effects, and significantly inhibits tumor growth (Snima et al., 2012; Anitha et al., 2014). Complement regulatory protein CD55 (Mamidi et al., 2013) is a decay accelerating factor, presents an anchored Glycosylphosphatidylinositol (GPI) moiety on cell membranes, and it is often highly expressed on the surfaces of tumor cells. This protein may be a target which can make tumor cells escape from autoimmune monitoring and immunotherapy. We synthesized CD55sp (Li et al., 2018) to target drug formulations to CD55 molecules highly expressed on the surfaces of cervical cancer HeLa cells.

In this study, C-PC/CMC-CD55sp nanospheres were constructed based on the antitumor activity of C-PC, the biocompatibility of CMC, and the targeting properties of CD55sp. These nanospheres were evaluated for antitumor efficacy, and the mechanisms of action were characterized *in vivo* and *in vitro*.

## Materials and Methods

### Materials

HeLa cells were purchased from Zhongqiao Xinzhou Biotechnology (Shanghai, China). NU/NU nude mice were purchased from Weitong Lihua Laboratory Animal Technology (Beijing, China). C-PC was purchased from Binmei Biotechnology (Taizhou, China). CMC was purchased from Honghai Biotechnology (Qingdao, China). CD55sp (QVNGLGERSQQM) was purchased from Gill Biochem (Shanghai, China). Cell Counting Kit-8 (CCK-8) was purchased from Biosharp (Hefei, China). Annexin V-FITC/PI apoptosis detection kit and TUNEL kit were purchased from Yeasen Biotechnology (Shanghai, China). Enzyme-linked immunosorbent assay kits were purchased from Gene Mei (Wuhan, China). Rabbit anti-human Bcl-2, caspase 3, and goat anti-rabbit IgG antibody were purchased from Abcam (UK). A laser particle size analyzer (Nano ZS90) was purchased from Malvern Instruments (UK). A full-function micropore detector was purchased from BIO-TEK (USA). A flow cytometer (Accuri C6) was purchased from Bidi Medical Devices (Shanghai, China). A fluorescence microscope (DP80) was purchased from Olympus (Japan). Powerpac Basic was purchased from BIO-RAD (USA). Vilber Fusion Solo Chemiluminescence Imaging System (4S) was purchased from Vilber (France).

### Synthesis of C-PC/CMC-CD55sp Nanospheres

Using CaCl_2_ (1.5 mg/ml) as a cross-linking agent, C-PC/CMC nanospheres were spontaneously constructed by encapsulating C-PC (1 mg/ml) with CMC (2 mg/ml) at 4°C for 30 min in the dark. EDC (2 mg/ml) was added to the C-PC/CMC nanospheres solution. The solution was adjusted to pH 5.6 and stirred at 4°C in the dark for 1 h. C-PC/CMC-CD55sp nanospheres were prepared by combining CD55sp (0.1 mg/ml) overnight at 4°C in the dark (Wang et al., 2017). Encapsulation Efficiency (EE) and Loading Efficiency (LE) of C-PC in nanospheres were calculated as follows (Yang et al., 2017):

$$\text{EE }=\frac{\text{weight of C}-\text{PC in the nanospheres}}{\text{ initial weight of C}-\text{PC}}\;\times \;100\%$$

$$\text{LE }=\;\frac{\text{weight of C}-\text{PC in the nanospheres}}{\text{weight of the nanospheres }}\;\times \;100\%$$

Nanospheres were evaluated using FTIR spectra across the range of 500–4,000 cm^-1^ (Abbas et al., 2018; Dong et al., 2018). Nanospheres morphology was observed using a transmission electron microscope (Shi et al., 2016; Nantachit et al., 2017; Tao et al., 2018). Zeta potential (surface charge) and particle size (nanospheres size) were measured using a laser particle size analyzer at 25°C (Zhang et al., 2015; Ahmad et al., 2018; Panchu et al., 2018; von Halling Laier et al., 2018).

### Cell Culture and Experimental Grouping

Cell lines were cultured in MEM supplemented with 10% newborn calf serum and incubated in a humid incubator at 37°C and 5% CO_2_. Cells were divided into the following four groups: Control (no drug treatment); C-PC (treated with C-PC); C-PC/CMC (treated with C-PC/CMC nanospheres); and C-PC/CMC-CD55sp (treated with C-PC/CMC-CD55sp nanospheres). The IC50 values in the C-PC/CMC-CD55sp were calculated after incubation for 24 h, and these values were used as the drug concentration for all subsequent cell experiments.

### Cell Counting Kit-8

Cells were treated with different concentrations of C-PC, C-PC/CMC, and C-PC/CMC-CD55sp in a 96-well plate, and incubated for 24 h in CO_2_ incubator. After the medium was aspirated, and the cells were incubated with CCK-8 mixture for 2 h. Measurement of absorbance was carried out at wavelength of 450 nm using a full-function micropore detector (Liu et al., 2013).

### Flow Cytometry

In 6-well plates, cells were treated with C-PC, C-PC/CMC, or C-PC/CMC-CD55sp. The cells were digested with pancreatin without EDTA, collected, centrifuged, and washed with PBS. After resuspended in 1× binding buffer, the cells were incubated with annexin V-FITC and PI staining solution at room temperature for 15 min in the dark. Then the solutions were mixed with an appropriate amount of 1× binding buffer, and placed on ice. The samples were evaluated using ﬂow cytometry within 1 h (Liang et al., 2017; Murata et al., 2018; Sui et al., 2018b; Zhao et al., 2018).

### Laser Confocal Microscopy

Cells were pretreated with C-PC, C-PC/CMC, or C-PC/CMC-CD55sp in 24-well plates, then incubated at 37°C in a 5% CO_2_ incubator for 24 h. Four percent paraformaldehyde were added to fix the cells. The cells were stained with DAPI solution for 10 min. After washing with PBS, cell slides were treated with antifade mounting medium and visualized using laser confocal microscopy (Costa Lima and Reis, 2015; Srivastav et al., 2019).

### TUNEL Analysis

Cells were pretreated with C-PC, C-PC/CMC and C-PC/CMC-CD55sp in 24-well plates. The cells were then fixed using 4% paraformaldehyde. After processed successively by Proteinase K solution and 1× equilibration buffer, the cells were incubated with excess TDT incubation buffer at 37°C for 1 h in the dark. Then cell nuclei were stained using DAPI. After washing with deionized water, the samples soaked in the PBS were immediately analyzed using a ﬂuorescence microscope (Sui et al., 2018b).

### Electron Microscopy

In six-well plates, cells were treated with Control solution and C-PC/CMC-CD55sp, then scraped off the wells. After centrifugation, the precipitates were fixed using glutaraldehyde, and the cells were visualized using transmission electron microscopy (Guardiola et al., 2017).

### Western Blot Analysis

In 6-well plates, cells were divided into C-PC, C-PC/CMC, and C-PC/CMC-CD55sp. After cells were lysed, the supernatants were collected. Then total protein concentrations were measured using the BCA assay. Proteins were separated using SDS-PAGE and transferred to PVDF membranes, and blocked with 5% skim milk for 1 h. The membranes were immunoblotted with the target primary antibodies at 4°C overnight. After washing with PBST, the membranes were treated with HRP-conjugated secondary antibody at room temperature for 1 h. Bands were visualized using chemiluminescence, and gray value analysis of the bands was performed using ImageJ software (Saleh et al., 2014; Lin et al., 2016).

### Nude Mouse Tumor Model

Mice were inoculated with a 0.2-ml subcutaneous injection of HeLa cell suspension (1 × 10^7^ cells/ml) under the armpit of the right forelimb. After tumor formation, the mice were also separated into control, C-PC, C-PC/CMC and C-PC/CMC-CD55sp. We injected C-PC/CMC-CD55sp into the tail veins of the mice, and the fluorescence intensity of the tumors was observed using small animal imaging system at 6 and 24 h. After 24 h, the mice were sacrificed and the fluorescence intensities of the heart, liver, spleen, kidney, and tumors were observed using small animal imaging system (Yeh et al., 2016; Li C. et al., 2017; Li W. et al., 2017; Wan et al., 2017; Song et al., 2018). An additional five mice per group were injected with doses based on body weight, and the IC50 values determined in the C-PC/CMC-CD55sp were used as the drug concentrations for each group. Mice were injected once every 2 days, and sacrificed after 20 days. Tumors and serum were collected for subsequent experiments (Li et al., 2013; Gao et al., 2016; Yeh et al., 2016; Wang et al., 2017; Sui et al., 2018a).

### Hematoxylin and Eosin Staining

For histological studies, tumor tissue samples (1 cm by 1 cm) were fixed in 10% formalin for a week to prepare paraffinized blocks by routine histological techniques. The 6-µm paraffinized sections were dewaxed and hydrated, then rinsed three times with PBS (3 min each). The samples were stained with hematoxylin for 5 min, faded with 1% HCl, and rinsed six times with ddH_2_O (5 min each). The samples were stained blue using a lithium carbonate saturated solution for 1 to 2 min, then rinsed three times with double-distilled water (5 min each). Stain separation was performed using 80% alcohol followed by rinsing three times with double-distilled water (5 min each). The sample were then stained with eosin for 5 min, and rinsed three times with double-distilled water (5 min each). The sections were then dehydrated, hyalinized, and mounting using resin. Field by field assessments of tissue morphology were performed using a light microscope (Abedpour et al., 2018; Baig et al., 2018; Liu et al., 2018; Wang et al., 2018; Gao et al., 2018).

### Enzyme-Linked Immunosorbent Assay

We used enzyme-linked immunosorbent assay (ELISA) kits to determine levels of interleukin-6 (IL-6), tumor necrosis factor-α (TNF-α), and transforming growth factor β (TGF-β) (Chen et al., 2004; Yang et al., 2010; Shi et al, 2018). Serum was collected and tested according to the ELISA kit manufacturer's instructions. Analysis was performed within 15 min after adding the stop solution.

### Statistical Analysis

Statistical analyses were performed using Prism 5.0 (GraphPad Software, Inc., La Jolla, CA, USA). Results are expressed as the mean ± SEM of three or more observations (as indicated in each experiment). The mean values for biochemical data from two groups were compared using two-tailed Student's *t*-tests. *P*-values less than 0.05 were considered statistically significant.

## Results

### Encapsulation Efficiency and Loading Efficiency

**Figure 1:**
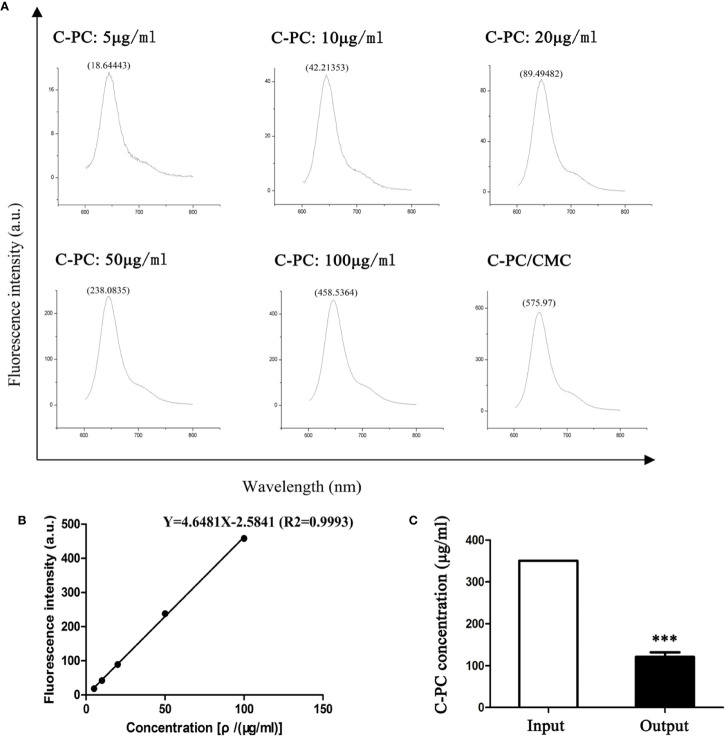
Encapsulation Efficiency (EE) and Loading Efficiency (LE) of C-Phycocyanin (C-PC)/Carboxymethyl Chitosan (CMC) nanospheres. **(A)** Fluorescence spectrophotometry assay. The horizontal axis represents the wavelength (nm) and the vertical axis represents the fluorescence intensity (a.u.). The fluorescence intensity in response to 5, 10, 20, 50, and 100 μg/ml C-PC at 644 nm was 18.64443, 42.21353, 89.49482, 238.0835, and 458.5364, respectively. The fluorescence intensity of the supernatant of the C-PC/CMC solution was 575.97. **(B)** Standard curve. The horizontal axis represents the concentration [ρ/(μg/ml)] and the vertical axis represents the fluorescence intensity (a.u.). The concentration was linearly related to the fluorescence intensity, and the standard curve was used to determine the concentration of the supernatant of the C-PC/CMC solution. **(C)** Supernatant analysis. The initial concentration of C-PC was used as the input, and the concentration of the supernatant of the C-PC/CMC solution was the output. Results are expressed as the mean ± SEM (n = 3). ^***^P < 0.001.

The standard curve equation for C-PC concentration and fluorescence intensity was Y = 4.6481X-2.5841 (r^2^ = 0.9993), and was linear across the range of 5–100 μg/ml (**Figures 1A, B**). The prepared C-PC/CMC nanospheres solution was centrifuged, and the fluorescence intensity of the supernatant was measured to determine the concentration. The EE was 65%. The samples were then precipitated by centrifugation, and the precipitates were freeze-dried. The net weights of the precipitates were determined using an electronic balance to determine the weights of the nanospheres. The LE was 20% (**Figure 1C**).

### Characteristics of Nanospheres

**Figure 2:**
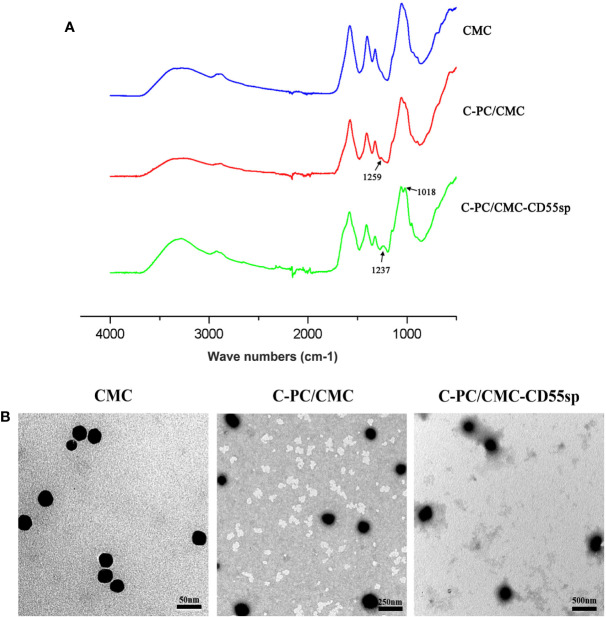
Characterization of nanospheres. **(A)** FTIR spectra for Carboxymethyl Chitosan (CMC), C-Phycocyanin (C-PC)/CMC, and C-PC/CMC-CD55sp. **(B)** Morphology was determined using transmission electron microscopy. CMC, scale bars, 50 nm; C-PC/CMC, scale bars, 250 nm; C-PC/CMC-CD55sp, scale bars, 500 nm.

In **Figure 2A**, based on FTIR spectra, we find that the peak situated at 1259 cm^−1^ in C-PC/CMC nanospheres related to C-O stretching turned to wave number 1237 cm^−1^ and 1018 cm^−1^ in C-PC/CMC-CD55sp nanospheres, and which may attribute to C-PC/CMC nanospheres modified with CD55sp resulted in the shifting and increase of peaks. In **Figure 2B**, the nanospheres were spherical and uniformly dispersed, as determined using electron microscopy. The nanospheres were dehydrated as a result of drying, which resulted in a bias in determination of particle size using electron microscopy. The relative sizes of nanospheres were compared using electron microscopy to confirm that the nanospheres contained drug. Compared with CMC nanospheres, the relative diameter of C-PC/CMC nanospheres was larger. This larger diameter may have been due to encapsulation of C-PC within the CMC nanospheres. The larger relative diameter of C-PC/CMC-CD55sp nanospheres may have been due to conjugation of CD55sp to the surface of C-PC/CMC nanospheres. As shown in **Table 1**, the particle sizes of CMC nanospheres, C-PC/CMC nanospheres, and C-PC/CMC-CD55sp nanospheres were 160.5 ± 48.06 nm, 146.6 ± 53.3 nm, and 258.9 ± 40.505 nm, respectively. The particle size of C-PC/CMC nanospheres was lower than that of CMC nanospheres, which may have been due to tighter internal bonds. Conjugation of CD55sp to the surface of C-PC/CMC nanospheres may have resulted in increased particle size. The zeta potentials of CMC nanospheres, C-PC/CMC nanospheres, and C-PC/CMC-CD55sp nanospheres were -7.66 ± 3.83 mV, -19.7 ± 2.53 mV, and -13.1 ± 3.28 mV, respectively. These values indicated that CMC and C-PC were negatively charged, and CD55sp was positively charged.

**Table 1:** The sizes and zeta potentials of the developed nanospheres.

Nanospheres	Size (d.nm)	Zeta potential (mV)
CMC	160.5 ± 48.06	-7.66 ± 3.83
C-PC/CMC	146.6 ± 53.3	-19.7 ± 2.53
C-PC/CMC-CD55sp	258.9 ± 40.505	-13.1 ± 3.28

### Targeting Ability of Nanospheres

**Figure 3:**
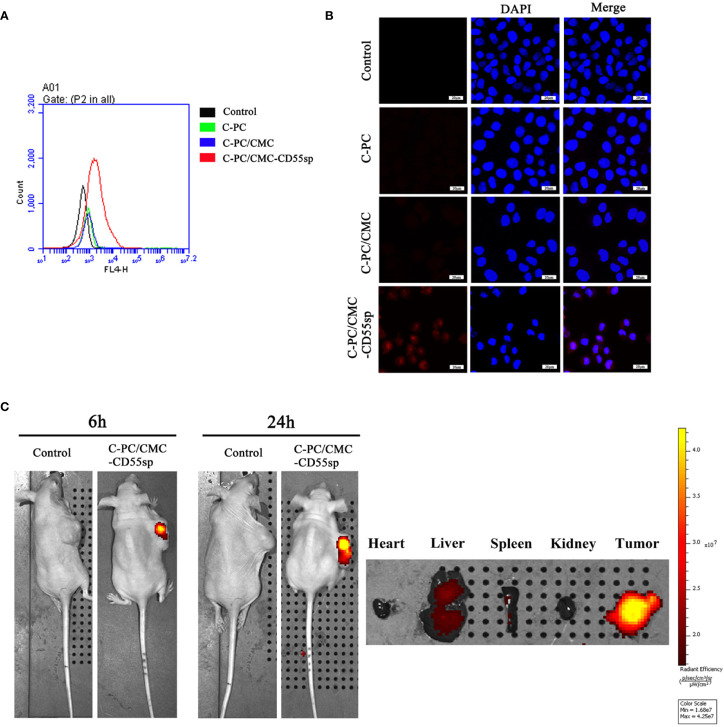
Determination of targeting effects using flow cytometry, laser confocal microscopy, and imaging. **(A)** Flow cytometry analysis. The fluorescence intensity of HeLa cells was determined using flow cytometry. The horizontal axis represents fluorescence intensity. Fluorescence intensity represented the targeting ability of drugs. **(B)** Laser confocal microscopy. Fluorescence intensity of HeLa cells was determined using laser confocal. Blue fluorescence represents nuclei, and red fluorescence represents targeting ability. **(C)** Imaging. Fluorescence intensity in tumor tissues and organs (heart, liver, spleen, and kidney) was detected using small animal imaging system. The color scale represents fluorescence intensity. Fluorescence intensity represents the targeting ability of drugs.

To investigate cellular uptake of nanospheres into HeLa cells, C-PC was used as a fluorescent marker. The fluorescence intensity was higher in the C-PC/CMC-CD55sp group than that in the other groups, as determined using flow cytometry and laser confocal microscopy, which indicated that this formulation was targeted to HeLa cells (**Figures 3A, B**). The results showed that the nanospheres formulation enhanced the internalization of drugs into HeLa cells. To evaluate the tissue distribution of nanospheres *in vivo*, the fluorescent marker C-PC was detected in a nude mouse model. The mice were injected with C-PC/CMC-CD55sp nanospheres *via* the tail vein, and fluorescence was observed 6 h after injection, and the fluorescence intensity was increased 24 h after injection. The nanospheres accumulated in the liver, the spleen, and the tumor, with most accumulation observed in the tumor. Accumulation of the nanospheres in the liver and the spleen may have been related to drug metabolism and the reticuloendothelial phagocytosis (**Figure 3C**). These results indicated that C-PC/CMC-CD55sp nanospheres were targeted to tumors, and that CD55sp may be an effective tumor targeting factor.

### Inhibition of Proliferation

**Figure 4:**
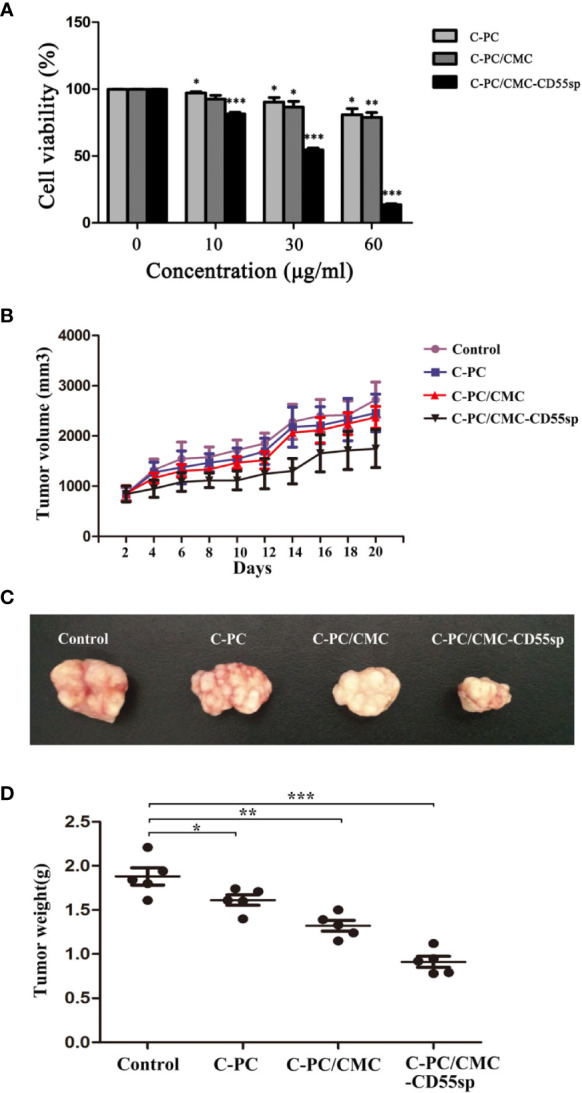
Inhibitory effects of drugs on cell proliferation *in vitro* and *in vivo*. **(A)** Cell Counting Kit-8 (CCK-8) assay. The x-axis represents drug concentration (0, 10, 30, and 60 μg/ml), and the y-axis represents cell activity, as determined using the CCK-8 assay. Results are expressed as the mean ± SEM (n = 3). ^*^P < 0.05, ^**^P < 0.01, ^***^P < 0.001. **(B)** Tumor volume. The horizontal axis represents days and the vertical axis represents tumor volume (mm^3^). **(C)** Tumor size. **(D)** Tumor weights. The vertical axis represents tumor weights (g). Results are expressed as the mean ± SEM (n = 5). ^*^P < 0.05, ^**^P < 0.01, ^***^P < 0.001.

In **Figure 4A**, HeLa cells proliferation decreased in a dose-dependent manner in response to treatment with C-PC, C-PC/CMC, and C-PC/CMC-CD55sp. Furthermore, treatment with C-PC/CMC-CD55sp inhibited proliferation of HeLa cells to a greater extent than the other formulations. The IC50 value for C-PC/CMC-CD55sp in HeLa cells was about 40 μg/ml. We also evaluated the antitumor effects of the nanospheres in tumor-bearing nude mice. After 20 days of observation and measurement, no nude mice in the C-PC, C-PC/CMC, or C-PC/CMC-CD55sp exhibited weight loss or showed signs of significant toxicity, and all animals survived to the end of the experiment. As shown in **Figure 4B**, tumor growth rate was inhibited by each of the drugs, and C-PC/CMC-CD55sp inhibited tumor growth to the greatest extent. The size and weight of the tumors were measured following sacrifice, and the results were consistent with those for tumor growth (**Figures 4C, D**). These results showed that C-PC, C-PC/CMC, and C-PC/CMC-CD55sp inhibited tumor growth, and C-PC/CMC-CD55sp induced the strongest inhibitory effect.

### Apoptosis

**Figure 5:**
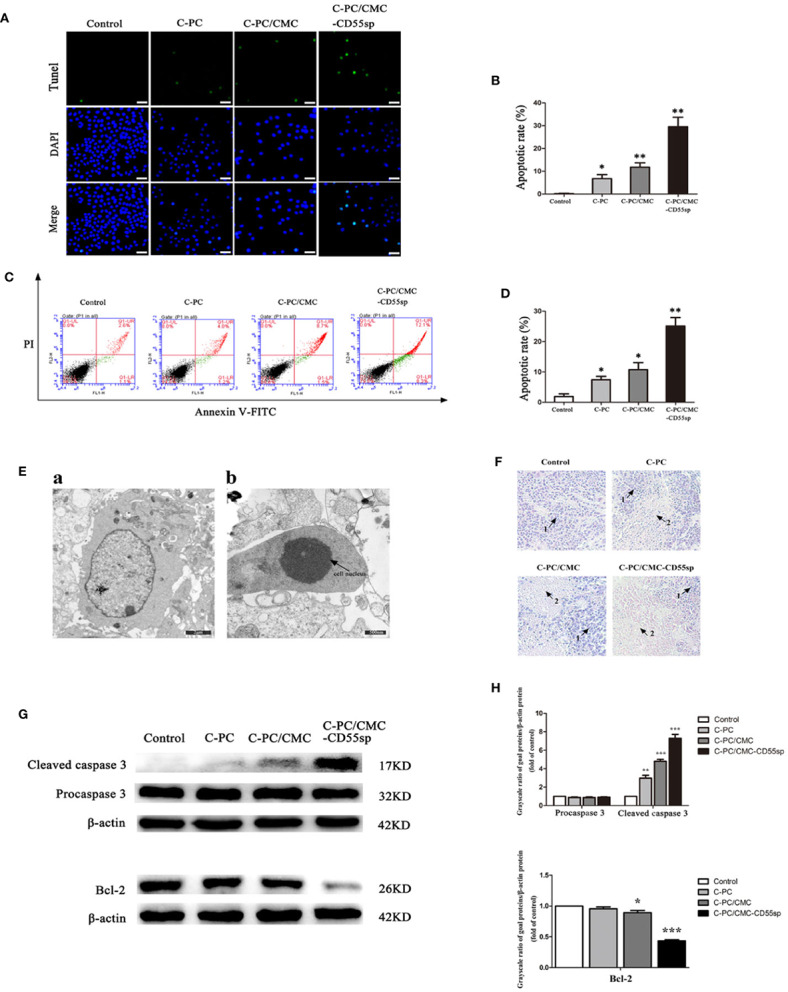
Drug-induced apoptosis. **(A, B)** TUNEL assay. Blue fluorescence indicates nuclei, and green fluorescence indicates apoptotic cells. The percentage of apoptotic cells (%) was equal to the ratio of the number of stained cells to the total number of cells. Results are expressed as the mean ± SEM (n = 3). ^*^P < 0.05, ^**^P < 0.01. Scale bars, 20 μm. **(C, D)** Flow cytometry analysis. Apoptosis was evaluated using an annexin V-FITC and PI apoptosis detection kit. The apoptosis rates of cells were determined, and C-Phycocyanin (C-PC)/CMC-CD55sp nanospheres induced apoptosis to the greatest extent. Results are expressed as the mean ± SEM (n = 3). ^*^P < 0.05, ^**^P < 0.01. **(E)** Electron microscopy. a. Normal HeLa cells. Scale bars, 2 μm; b. Apoptotic HeLa cells. Scale bars, 500 nm. Arrows indicate nuclei. **(F)** Hematoxylin and eosin staining. (1) Normal HeLa cells; (2) apoptotic HeLa cells. **(G, H)** Western blot. Protein levels were normalized to β-actin. Results are expressed as the mean ± SEM (n = 3). ^*^P < 0.05, ^**^P < 0.01, ^***^P < 0.001.

Apoptosis is a key indicator of the antitumor ability of drugs. We found that C-PC, C-PC/CMC, and C-PC/CMC-CD55sp increased apoptosis in HeLa cells, and C-PC/CMC-CD55sp induced apoptosis to the greatest extent by TUNEL assay (**Figures 5A, B**) and flow cytometry analysis (**Figures 5C, D**). In **Figure 5E** a, ultrastructural analysis of untreated HeLa cells was used to characterize the normal morphology of the control group. In **Figure 5E** b, cells treated with C-PC/CMC-CD55sp showed apoptotic morphology, which included cytoplasm concentration, decreased cell volume, nuclear shrinkage and deepened staining, and disappearance of microvilli. As shown in **Figure 5F**, tumor tissue sections were prepared from tumor-bearing nude mice to further evaluate apoptosis. In the Control group, the tumor cells indicated by arrow 1 were closely aligned, with clear margins and normal karyotypes. In the three treatment groups, the tumor cells indicated by arrow 2 were dispersed, and showed characteristic apoptotic changes, such as nuclear shrinkage, nuclear rupture, and nuclear dissolution. Apoptosis is tightly regulated by apoptosis-related proteins such as Bcl-2 and caspase 3. **Figures 5G, H** showed that the levels of Bcl-2 protein and cleaved caspase 3 were lower in response to treatment with nanospheres, and C-PC/CMC-CD55sp induced the most pronounced changes. These results showed that C-PC/CMC-CD55sp induced apoptosis by altering the levels of apoptosis-related proteins.

### Immunoregulation

**Figure 6:**
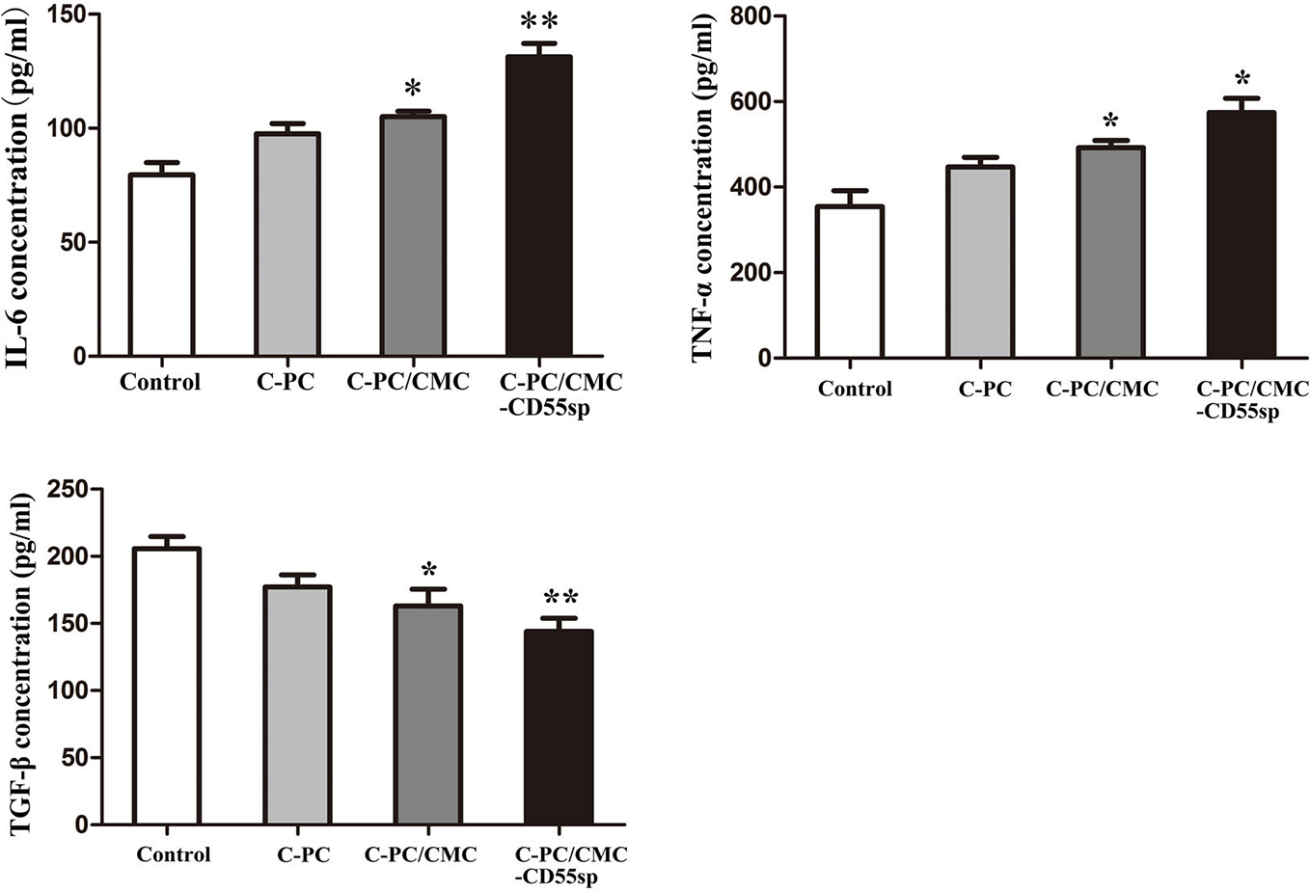
Levels of interleukin-6 (IL-6), tumor necrosis factor-α (TNF-α), and transforming growth factor β (TGF-β) in nude mice. Enzyme-linked immunosorbent assay. The vertical axis represents concentration (pg/ml). Results are expressed as the mean ± SEM (n = 3). ^*^P<0.05, ^**^P<0.01.

To evaluate the role of immune response in tumor killing, the levels of IL-6, TNF-α, and TGF-β were measured in mouse serum using ELISA. The levels of IL-6 and TNF-α in the C-PC, C-PC/CMC, and C-PC/CMC-CD55sp groups were higher than those in the Control group. In contrast, the levels of TGF-β were lower in the treated groups than those in the Control group, and C-PC/CMC-CD55sp induced the strongest effect (**Figure 6**). These results indicated that C-PC/CMC-CD55sp stimulated secretion of IL-6 and TNF-α, and reduced the expression of TGF-β, which might indicate that tumor cells death occurred through modulation of the immune response.

## Discussion

**Figure 7:**
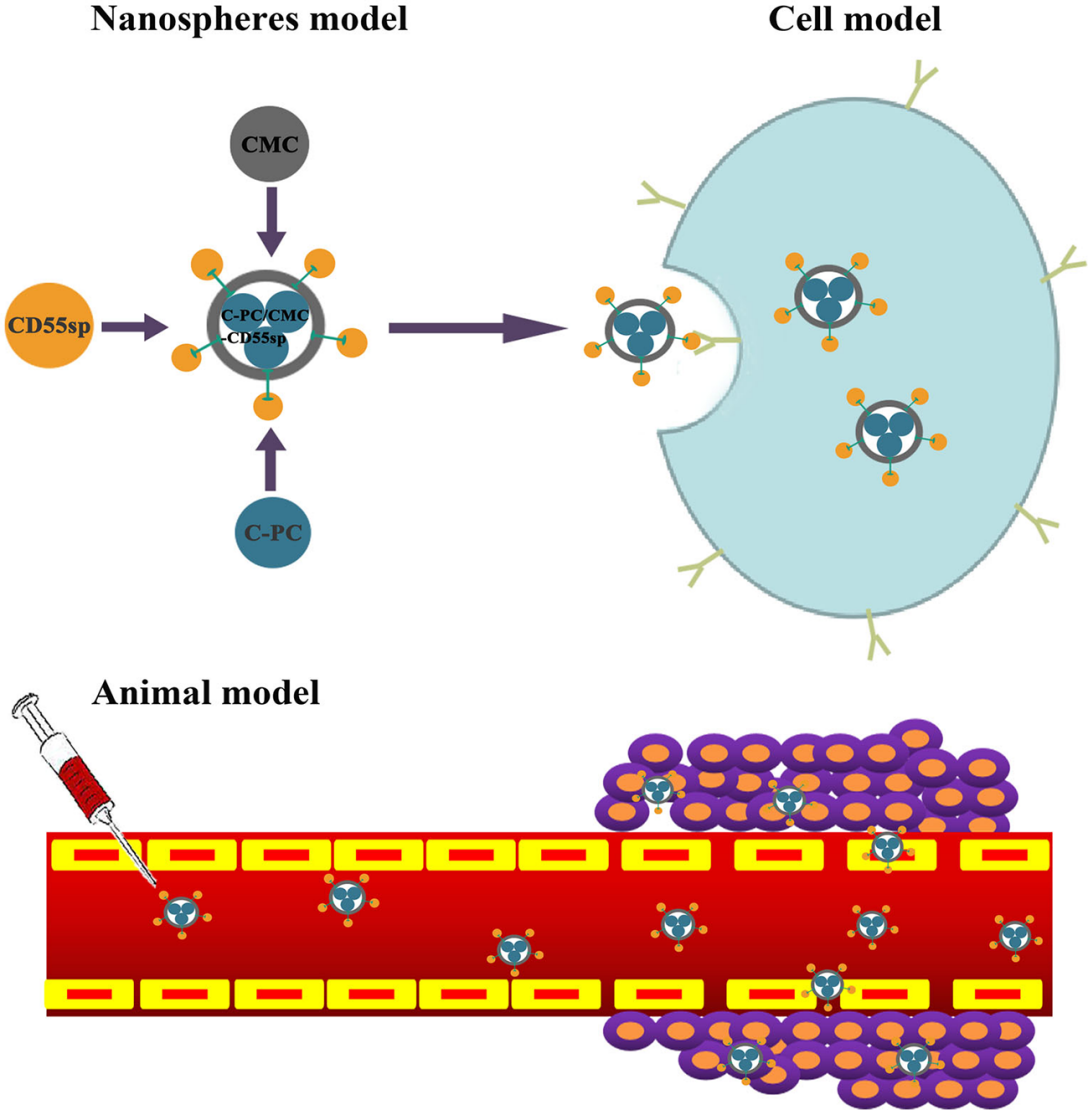
A model for C-PC/CMC-CD55sp nanospheres in targeting tumor cells *in vivo* and *in intro*.

Cervical cancer is among the most common types of gynecological malignant tumors (Torre et al., 2015). Base on the epidemiological studies, there are about 500,000 new cases of cervical cancer globally each year (Torre et al., 2015). Li B. et al. (2006) showed that release of cytochrome C from mitochondria to the cytoplasm following C-PC treatment of HeLa cells was associated with apoptosis. Another study showed that CMC-co-poly (AA) had potential for targeted delivery of various antitumor drugs (Sharif et al., 2017). Li et al. (2018) also showed that CD55sp could bind to CD55 molecules on the surface of HeLa cells as a ligand peptide. Therefore, we constructed novel C-PC/CMC-CD55sp nanospheres with C-PC included as an anticancer drug, CMC as a carrier, and CD55sp as a targeting peptide. Targeted inhibition of proliferation and apoptosis were evaluated in HeLa cells (**Figure 7**).

The EE and LE of the nanospheres were determined (Yang et al., 2017). The nanospheres were characterized using FTIR (Abbas et al., 2018; Dong et al., 2018), electron microscopy (Shi et al., 2016; Nantachit et al., 2017; Tao et al., 2018), and laser particle size analysis (Couvreur et al., 2002; Zhang et al., 2015; Shi et al., 2016; Ahmad et al., 2018; Panchu et al., 2018; von Halling Laier et al., 2018). The results showed that C-PC/CMC-CD55sp nanospheres were successfully constructed.

Flow cytometry (Ying et al., 2018), laser confocal microscopy (Costa Lima and Reis, 2015; Srivastav et al., 2019), and small animal imaging system (Yeh et al., 2016; Li C. et al., 2017; Li W. et al., 2017; Wan et al., 2017; Song et al., 2018) showed that C-PC/CMC-CD55sp nanospheres were targeted to tumor cells. These results showed that the developed nanospheres targeted HeLa cells *in vitro* and *in vivo* in a tumor-bearing mouse model.

Cell viability was analyzed as the proportion of healthy cells in a sample, and proliferation has been shown to be an important parameter for understanding the pathways involved in cell survival or death after treatment (Adan et al., 2016). Generally, methods used to determine cell viability have also been used to determine cell proliferation (Adan et al., 2016). Furthermore, cell proliferation assays have been generally used for drug screening to determine whether the test molecules had induced the desired effects (Adan et al., 2016). In our study, CCK-8 was used to evaluated the effects of C-PC, C-PC/CMC, and C-PC/CMC-CD55sp on HeLa cell proliferation. The results showed that C-PC/CMC-CD55sp induced the strongest antitumor effect.

Tumorigenesis results from disruption of the balance between proliferation and apoptosis, and apoptotic signal transduction is a key factor in apoptosis. To detect nuclear DNA cleaved by activated DNases during late stages of apoptosis, TUNEL staining is typically used (Fayzullina and Martin, 2014). Flow cytometry can be used to identify apoptotic cells through binding of dye to phosphatidylserine on the cell surface of early apoptosis, and through binding of dyes to DNA of late apoptotic or necrotic cells (Wlodkowic et al., 2011; Jiang et al., 2017). Dynamic changes in compaction of nuclear chromatin are characteristic of apoptosis (Wyllie et al., 1984). During apoptosis, chromatin undergoes a phase change from a heterogeneous, genetically active network to an inert highly condensed fragmented form (Maruyama et al., 2001; Tone et al., 2007). Cell morphology, size, and changes in organelles can also be used to identify apoptotic cells (Taatjes et al., 2008). Apoptosis-related proteins such as the caspase 3 protease family (Hsu et al., 2018; Safavi et al., 2018) and the antiapoptotic protein Bcl-2 (Pihan et al., 2017; Beberok et al., 2018) play key roles in apoptosis. Using western blot, we determined the expression of cleaved caspase 3 and Bcl-2 to evaluate apoptosis in tumor cells. The results showed that C-PC/CMC-CD55sp induced apoptosis of HeLa cells to the greatest extent, which resulted in strong antitumor effects.

We detected cytokines such IL-6, TNF-α, and TGF-β in mouse serum, and showed that the developed nanospheres induced an immune response, which may have regulated tumor killing. IL-6 signaling been shown to inhibit tumor growth by mobilizing antitumor T cell immune responses (Fisher et al., 2014). IL-6, which is produced by dendritic cells in lymph nodes, has been shown to impact activation, expansion, survival, and polarization of T cells during immune responses (Hope et al., 1995). IL-6 may participate in modulation of the T cell immune response, resulting in a shift from a suppressive to a responsive state that could promote antitumor activity. Furthermore, IL-6 has been shown to play an important role in promoting T cell trafficking to lymph nodes and to tumor sites, where they become activated and exert cytotoxic effector activity (Chen et al., 2004; Appenheimer et al., 2007; Fisher et al., 2011; Fisher et al., 2014).

TNF-α is produced by monocytes and macrophages, and plays a role in cell survival, apoptosis-related inflammation, and immune activity (Shi et al., 2018). In addition, TNF-α has been shown to be an effective antitumor agent *in vitro* and *in vivo* through induction of tumor apoptosis and necrosis (Li M.O. et al., 2006). High loco-regional doses of TNF-α have been shown to induce hemorrhagic necrosis *via* selective destruction of tumor blood vessels and generation of specific T cell antitumor immunity (Lejeune, 2002; Balkwill, 2006).

TGF-β signaling plays an important role in promoting tumor initiation and progression, and its mechanisms include dysregulation of cyclin-dependent kinase inhibitors, alteration of cytoskeletal architecture, increased protease expression and extracellular matrix formation, decreased immune surveillance, and increased angiogenesis (Massague, 2008; Yang et al., 2010). Studies have shown that TGF-β had an adverse effect on antitumor immunity and inhibited host tumor immune surveillance (Li M.O. et al., 2006; Yang et al., 2010). Furthermore, TGF-β markedly suppressed the ‘cytotoxic program' of cytotoxic T lymphocytes, which have been shown that favor tumor progression (Thomas and Massague, 2005; Yang et al., 2010). Detection of IL-6, TNF-α, and TGF-β in blood of tumor-bearing nude mice showed that C-PC/CMC-CD55sp nanospheres induced an immune response that was associated with tumor growth inhibition.

## Conclusion

We successfully constructed C-PC/CMC-CD55sp nanospheres and confirmed their targeting properties. These nanospheres inhibited HeLa cell proliferation and promoted HeLa cell apoptosis *in vivo* and *in vitro*. Furthermore, these nanospheres inhibited tumor tissue growth through regulation of the immune response *in vivo* in nude mice.

## Data Availability Statement

The raw data supporting the conclusions of this article will be made available by the authors, without undue reservation.

## Ethics Statement

The animal study was reviewed and approved by Medical Ethics Committee of Affiliated Hospital of Qingdao University.

## Author Contributions

GL, XX, LJ, and BL designed the study, performed the experiments, analyzed and interpreted the data. HJ, FZ, and BJ wrote and refined the manuscript. JH, XD, and FY assisted in the completion of the project. All authors contributed to the article and approved the submitted version.

## Funding

This work was supported by grants from the National Natural Science Foundation of China (Nos. 81871231, 81471546, and 81001346), Youth Innovation and Technology Plan of Shandong Colleges (2019KJK016), and Science and Technology Project of Qingdao (No. 18-6-1-91-nsh).

## Conflict of Interest

The authors declare that the research was conducted in the absence of any commercial or financial relationships that could be construed as a potential conflict of interest.
